# A Miraculous Save: Gangrenous Bowel and Meckle’s Diverticulum With Acute Superior Mesenteric Artery Thrombosis

**DOI:** 10.7759/cureus.52947

**Published:** 2024-01-25

**Authors:** Abhilasha Bhargava, Pankaj Gharde, Harshal Tayade, Akash Inamdar

**Affiliations:** 1 General Surgery, Jawaharlal Nehru Medical College, Datta Meghe Institute of Higher Education and Research, Wardha, IND

**Keywords:** anastomotic leak after gastrointestinal surgery, resection and anastomosis, gangrenous meckels diverticulum, meckels diverticulum, small bowel obstruction, superior artery thrombus

## Abstract

The superior mesenteric artery (SMA) is vital for parts of the small intestine and ascending colon. Thrombosis of this major artery is a severe and potentially fatal condition involving the occlusion of the arterial vascular supply, causing ischemia predisposing to gangrene. Meckel's diverticulum is a congenital outpouching in the lower part of the small intestine. The condition of gangrenous meckels diverticulum is, therefore, even more limited. This study presents a unique case of a 45-year-old male with coexisting features of SMA thrombus with acute small bowel intestinal obstruction. During the study, the patient was found to have sickle cell anemia with an AS pattern, which may have predisposed the formation of a thrombus. The patient underwent sequential management of active thrombus by thrombolysis first, followed by resection anastomosis for gangrenous bowel. With precise monitoring and therapeutic care, the patient made a remarkable recovery. The condition possesses a high mortality rate. Prompt recognition and timely intervention in this case are of utmost significance.

## Introduction

The superior mesenteric artery (SMA) is a vital supply for parts of the small intestine and ascending colon. Thrombosis of this major artery is a severe and potentially fatal condition involving occlusion of the arterial vascular supply, causing ischemia predisposing to gangrene [1]. Meckel's diverticulum is a congenital outpouching in the lower part of the small intestine. It occurs in about 2% to 3% of the general population. The condition of gangrenous meckels diverticulum is therefore even more limited [2]. The condition possesses a high mortality rate. Acute superior mesenteric ischemia is defined as a sudden interruption of the vascularity of the small intestine, causing ischemia with inflammatory changes. If left untreated, it will lead to fatal intestinal necrosis. Even though it's an unusual cause of abdominal pain, it requires utmost attention since mortality has been reported at approximately 50% [1,2]. The hallmarks of contemporary care are prompt surgical intervention and early diagnosis, both of which are necessary to lower the entity's high death rate. The combination of recent imaging modalities with endovascular procedures may offer novel benefits in terms of prediction. We present a unique case of a 45-year-old male with coexisting features of SMA thrombus with acute small bowel intestinal obstruction. The patient underwent sequential management for active thrombus and resection of gangrenous bowel with a good outcome.

## Case presentation

**Figure 1:**
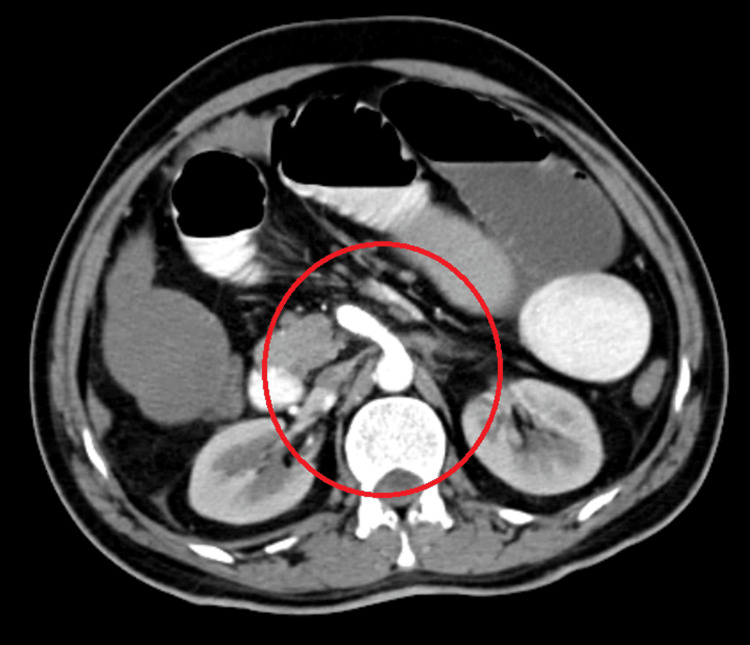
CECT abdomen pelvis: showing the stent in situ placed secondary to SMA occlusion CECT: contrast-enhanced computed tomography SMA: superior mesenteric artery

A 45-year-old male presented with complaints of obstipation and pain in the abdomen for three days. Abdominal pain was insidious in onset and gradual in progression, which was associated with high-grade fever, bilious vomiting, and abdominal distension. His general condition was poor with tachycardia of 120/min, tachypnea of 22/min, BP of 110/70 mmHG, and occasional fever spikes with a daily Ryle's tube collection of 500 mL, which was bilious in consistency. On admission, the patient presented with hypertension, tachycardia, and increased leukocyte count. Clinically, as per abdominal assessment, the patient presented with guarding, rigidity, distension with sluggish bowel sounds. On digital rectal examination, the rectum was empty with ballooning of the anal canal. He was a newly diagnosed case of sickle cell anemia with AS pattern with a significant history of pulmonary tuberculosis two years back, for which he completed a category 1 directly observed therapy (DOTS) regimen. Significantly, he had a history of chewing tobacco for 20 years. A contrast-enhanced computed tomography (CECT) scan report suggested a 3.1 x 1.0 cm acute thrombus in proximal SMA and dilated jejunal and proximal ileal loops up to 3.8 cm with collapsed large bowel loops. The patient was taken up for emergency thrombolysis of SMA by an interventional radiologist. Post-thrombolysis, the patient was started on anticoagulants and antiplatelet medications. A repeat CECT abdomen pelvis was done, suggesting partial thrombosis of the proximal SMA of 3 x 0.5 cm. It suggested that small bowel obstruction predominantly involved the jejunal and ileal loops with a transition point in the distal ileal loop with central mesenteric fat stranding. An erect abdominal radiograph showed multiple air-fluid levels in the small intestine. For the compromised lumen of SMA, the patient underwent emergency stenting where an 8 x 5.9 mm stent was deployed across the lesion involving SMA, which was confirmed with good flow in the angiogram (Figure 1).

**Figure 2:**
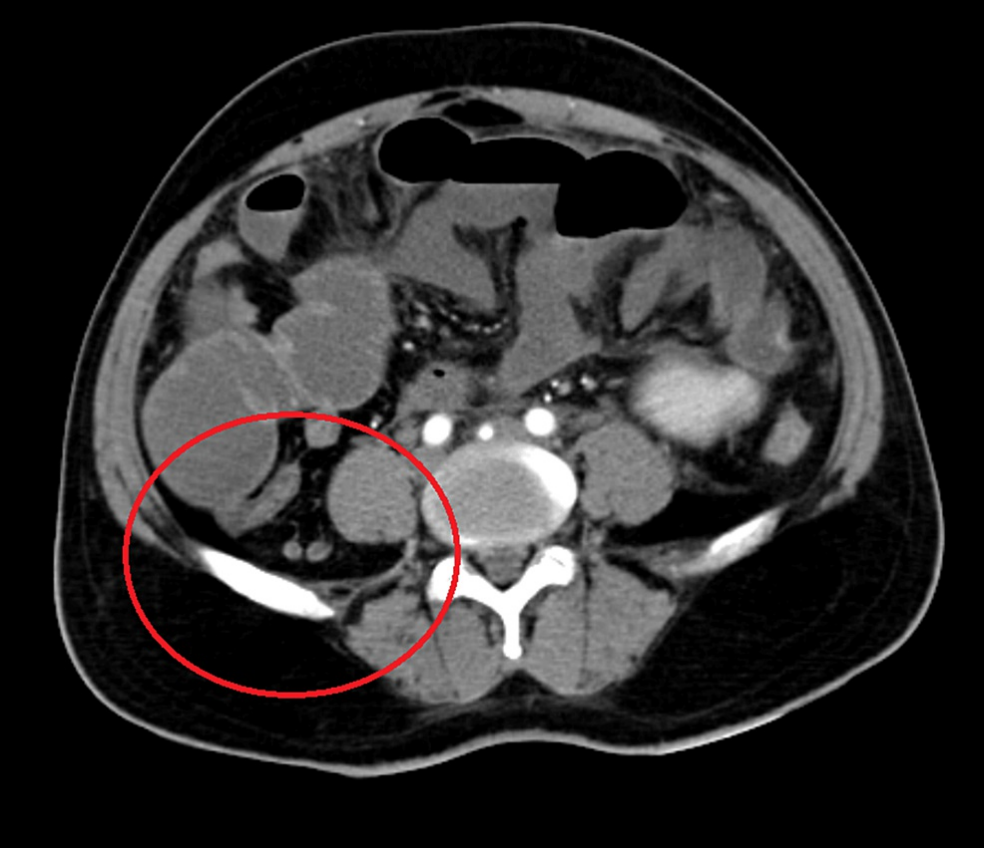
CECT abdomen-red encircled part showing transition point of intestinal obstruction in the distal ileal loop CECT: contrast-enhanced computed tomography

Post-stenting, the patient was started on thrombolytic agents and continued on anticoagulant. Vitally, the patient still presented with tachycardia of 140/min with a rigid abdomen yet reduced guarding because of intravenous analgesics. A repeat CECT abdomen pelvis revealed the persistence of dilated small bowel loops approximately 5 cm were seen with a transition point at the ileocaecal junction with query stricture and mild ascites with central mesenteric fat stranding (Figure 2).

**Figure 3:**
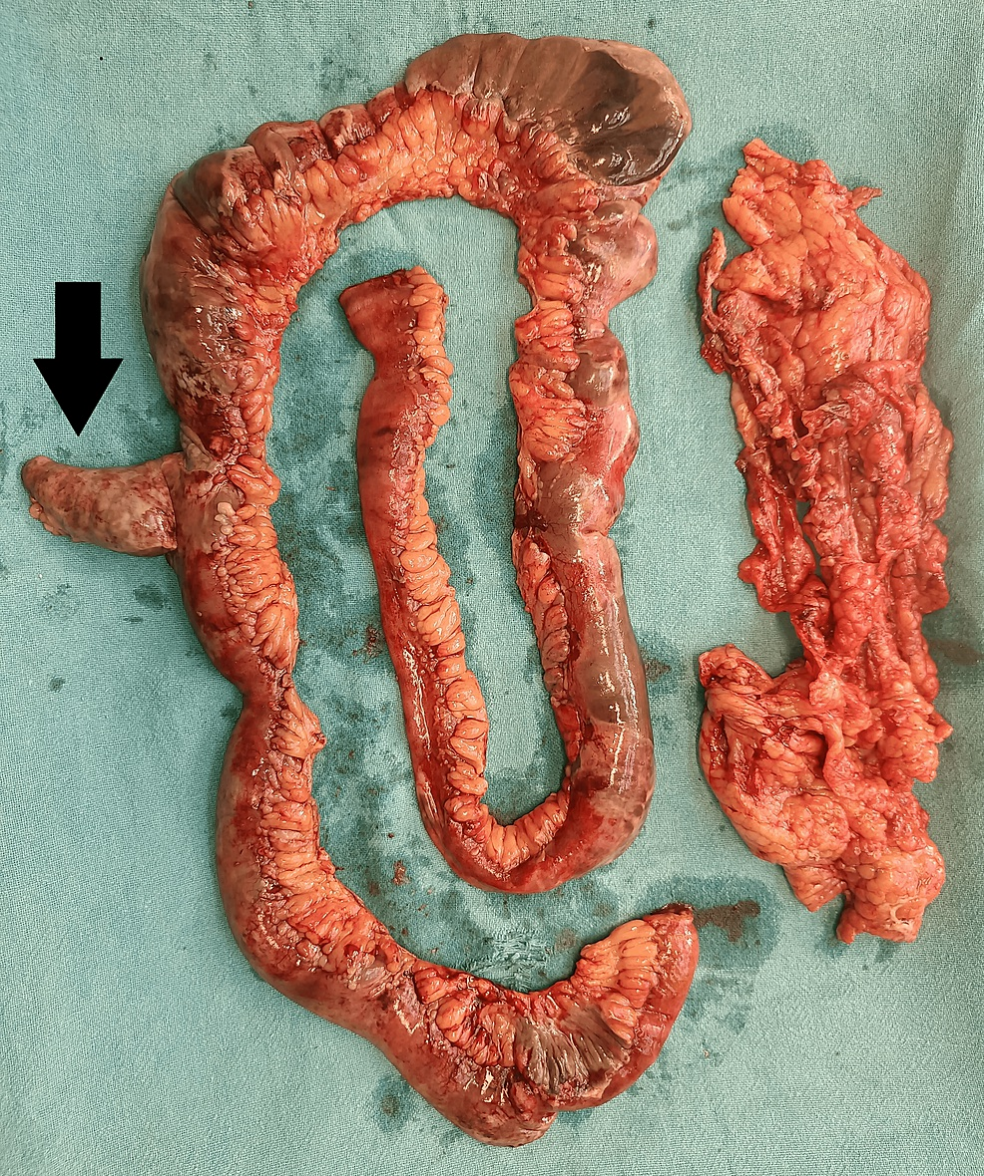
Clinical image showing the excised gangrenous small bowel with the black arrow depicting the gangrenous meckels diverticulum

Subsequently, the patient was taken for emergency exploratory laparotomy. Intraoperatively, there was evidence of 200 mL seropurulent, foul-smelling fluid on incising the peritoneum along with 130 cm gangrenous small bowel extending from mid jejunum to proximal ileum including gangrenous Meckel's diverticulum with collapsed large bowel. On tracing, the gangrenous bowel was found to be 230 cm distal to the duodenojejunal flexure and 150 cm proximal to the ileocaecal junction, with the Meckel's diverticulum being 80 cm proximal to the ileocaecal junction, while the colon was collapsed. There was the presence of impending gangrenous regions throughout the small bowel. The gangrenous segment of the small bowel was resected and sent for histopathology (Figure 3).

**Figure 4:**
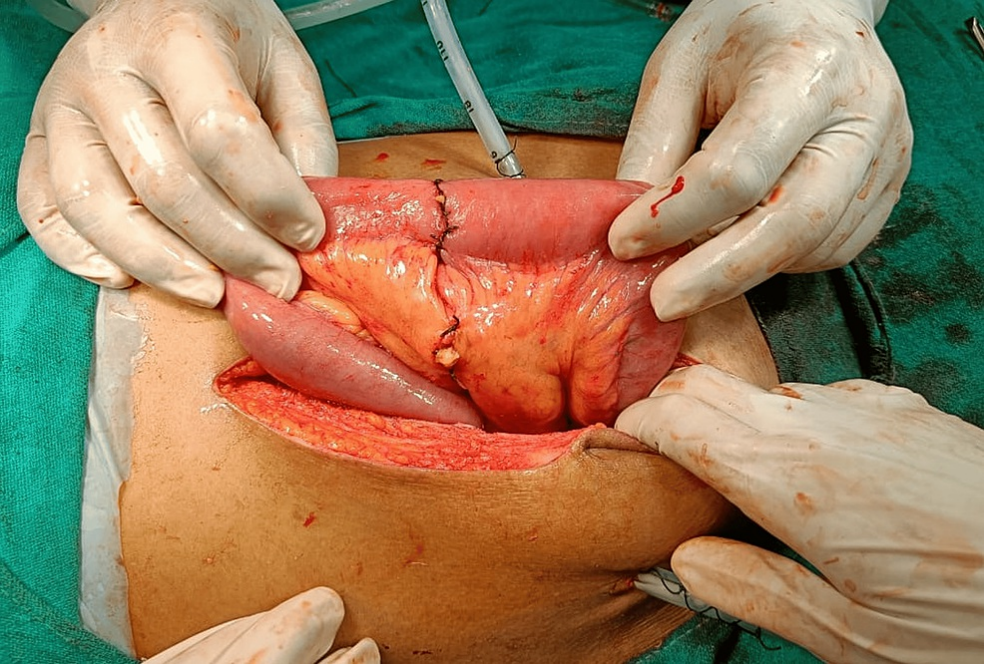
End to end jejuno-ileal anastomosis

A hand-sewn end-to-end jejunoileal two layered anastomosis was performed (Figure 4).

**Figure 5:**
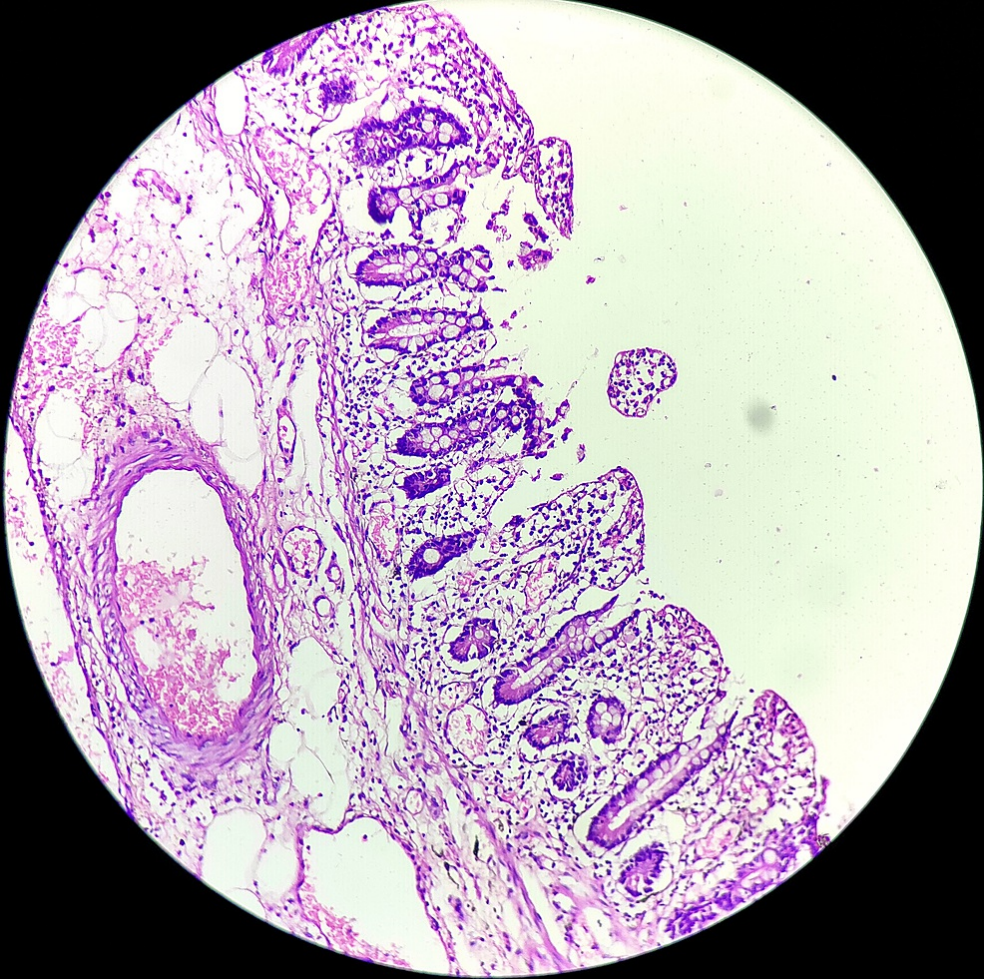
Histopathological slide depicting gangrenous small bowel

Thorough warm saline washes were given during the procedure to establish perfusion in the impending gangrenous regions. Postanastomosis drains in the Morrison's pouch and pelvis were placed before closing the laparotomy incision. Postoperatively, the patient was under ICU monitoring. On postoperative day three, there was feculent collection in the abdominal drain implying anastomotic leak. The patient underwent a revision anastomosis secondary to a leak because of a compromised blood supply during the first sitting. The patient continued to be on anticoagulants with higher antibiotic coverage with electrolyte corrections. The patient passed Bristol type 7 stools with flatus on postoperative day two of the second surgery. Eventually, the drain and Ryle's tube collection decreased. After meticulous monitoring and timely intervention, the patient’s condition improved remarkably. Tachycardia was settled along with biochemical and pathological investigations within normal range. The patient was ambulated with vigorous chest physiotherapy. A CECT abdomen was performed on postoperative day four, which was affirmative of no contrast leaks, with contrast reaching the sigmoid colon suggestive of functional anastomosis. On postoperative day six, the patient was shifted to the ward. The postoperative recovery was uneventful, with a healthy, healed suture site, and the patient was discharged on the 12th postoperative day. On follow-up, the patient is keeping well with no fresh complaints. The histopathology report for the excised gangrenous segment of the small intestine and Meckel's diverticulum was suggestive of liquefactive necrosis (Figure 5).

The management of SMA thrombus along with gangrenous bowel discussed in this case study emphasizes early identification and treatment to improve outcomes. The timely use of CECT to confirm the diagnosis, assessment of the need for revascularization to restore gangrenous bowel perfusion, removal of the necrotic intestine, and application of damage control techniques to permit evaluation of small bowel viability before a final anastomosis are important to note.

## Discussion

Meckel's diverticulum results from incomplete obliteration of the vitelline duct. Generally asymptomatic, various complications may occur like hemorrhage, intussusception, diverticulitis, perforation, and intestinal obstruction. There has been evidence of Meckel's diverticulum acting as a lead point for intestinal obstruction, which has the potential to cause further obstruction [2]. The average age of presentation is infancy, and presentation in adulthood is rare. Meckel's diverticulum occurs on the antimesenteric border of the ileum and in 90 percent of the cases within 90 cm from the ileocecal valve, although in our case it was 80 [1]. In our case, a rare intraoperative presentation of a gangrenous Meckel’s diverticulum, distal jejunum, and proximal ileum along with an SMA thrombus was observed, which was not reported earlier. The patient has a history of tobacco consumption for 20 years, which would have an increased tendency of the development of thrombus as the nicotine in tobacco causes a decrease in HDL, and an increase in LDL and VLDL cholesterol, favoring the formation of atherosclerotic plaques in the superior mesenteric arterial wall [3]. Depending on the size of the embolus, embolic occlusions may lodge more distally. Atherosclerosis, arrhythmias, cardiac illness, advanced age, abdominal cancer, and inflammatory bowel disease (IBD) are risk factors. According to estimates, the illness accounts for 0.1% of all hospital admissions overall. Severe ischemia damage caused by a disruption in the venous drainage of the bowel or disrupted blood supply typically leads to a perfusion insufficiency of the gastrointestinal tract. Such severe ischemia presents as intestinal blockage and stricture development, or less frequently as gangrene and perforation [4].

The majority of the small intestine and the ascending colon suddenly lose their primary blood supply as a result of SMA thrombosis, whether it is in situ or embolic. The percentage of the small bowel that is injured is directly related to how serious the lesion is. Generally, the severity of the damage increases with the proximity of the blockage. The length of ischemia, hypotension, and the presence (or lack) of collateral circulation are some other variables that may directly affect the severity of the injury. Two possible pathophysiologic descriptions behind the gangrenous changes could have been (i) ischemia because of active thrombus and (ii) post-inflammation. Even with timely investigation and intervention, an acute proximal SMA thrombus was caught early and was treated with thrombolysis followed by SMA stenting. Ischemia and gangrene developed because of the compromised blood supply [5]. However, the distended ileal and jejunal loop could also have been responsible for opposite pressure and compression effects on Meckel’s diverticulum, resulting in the intraoperative findings. Thus, a unique vicious cycle arising from the SMA thrombus causing small bowel gangrene, including Meckel's diverticulum, leading to ileojejunal obstruction, appears to be another pathophysiological event. Either of these mechanisms might have played the offending role in our case. Reports exist that there is a 50% chance of survival if diagnosis takes place within 24 hours. However, survival drops to 30% if the diagnosis is delayed beyond the 24-hour window [6]. The patient has a case of sickle cell trait with AS pattern further predisposes the formation of a thrombus. Sickle cell disease is a multisystemic disorder caused by a single point mutation in the sixth codon of the hemoglobin gene, leading to the substitution of valine for glutamic acid in the β-globin chain [7]. In a deoxygenated state, sickle hemoglobin (HbS) polymerizes, forming “sickled” erythrocytes, leading to a series of events causing protean manifestations [8]. The patient becomes at risk for thrombosis and thromboembolism because they exhibit features such as activated endothelium, venous stasis, and blood hypercoagulability. Revision anastomosis can be taken into consideration in clinically stable patients who don't have severe abdominal contamination or septic shock. Evidence of ischemia at the anastomosis or insufficient tissue integrity leads to recurrent anastomotic leak [9]. Revision of the anastomosis with proximal diversion has shown great results, but, in our case, we revised the anastomosis at the previous site itself. It has been established that the mortality and reoperation risk for both surgical treatment hypotheses are comparable [10]. The stability of the patient, the extent of the anastomotic defect, the level of contamination, and the integrity of the tissue all play a role in determining whether to resect, modify, or drain an anastomosis [11]. Thus, the majority of the decisions are determined by intraoperative discoveries.

## Conclusions

In conclusion, SMA thrombosis often has a bad prognosis. Patients with SMA thrombosis need timely clinical intervention, which leads to favorable outcomes. To improve the patient's prognosis, we presented this case report's conclusion, the significance of prompt intervention in addition to any necessary surgical treatments.
